# The prevalence of alcoholic and nonalcoholic fatty liver disease in adolescents and young adults in the United States: analysis of the NHANES database

**DOI:** 10.1186/s12876-022-02430-7

**Published:** 2022-07-30

**Authors:** Naim Alkhouri, Ashraf Almomani, Phuc Le, Julia Y. Payne, Imad Asaad, Celine Sakkal, Miriam Vos, Mazen Noureddin, Prabhat Kumar

**Affiliations:** 1 Hepatology and Liver Transplantation, Arizona Liver Health, 20201 W Fairview St, Chandler, AZ 85224 USA; 2 Cleveland Clinic Foundation, Cleveland, OH USA; 3 Cedar Sinai Medical Center, Los Angeles, CA USA; 4 Emory University School of Medicine, Atlanta, GA USA

**Keywords:** NAFLD, ALD, Alcohol, Fibroscan, Elastography, Fibrosis, Cirrhosis

## Abstract

**Background:**

The prevalence of fatty liver disease is potentially increasing in adolescents and young adults (AYAs) due to the obesity and alcohol pandemics. The aim of this study was to assess the prevalence of alcohol-associated fatty liver disease (ALD) and nonalcoholic fatty liver disease (NAFLD) in a representative U.S. cohort utilizing transient elastography to directly measure hepatic steatosis and suspected fibrosis.

**Methods:**

AYAs (age 15–39 years) with valid FibroScan^®^ measurements in the National Health and Nutrition Examination Survey (NHANES) database (2017–2018) were included in the analyses. Those with viral hepatitis, pregnancy, or ALT/AST > 500 U/L were excluded. The population was divided into those with excessive alcohol consumption (ALQ130) and those without. Controlled attenuation parameter (CAP) score ≥ 248 dB/m was used to identify suspected ALD and NAFLD. In those with evidence of ALD, the following cutoffs of liver stiffness measurement (LSM) were used for suspected fibrosis: F ≥ F2 at LSM ≥ 7.5 kPa and F ≥ F3 at ≥ 9.5 kPa, respectively. In those with suspected NAFLD, the following LSM cutoffs were used: F ≥ F2 at 6.1 and F ≥ F3 at ≥ 7.1, respectively. Cutoffs were chosen based on published literature to maximize sensitivity.

**Results:**

Comparing to those without, subjects with excessive alcohol consumption tended to be older (29.8 vs 28.5 years), have a higher BMI (29.3 vs 28.9 kg/m2), and be from a White ethnicity (65.3% vs. 55.4%). In subjects with excessive alcohol consumption, suspected ALD was present in 56.59% (95% CI 41.57–70.49). In those with suspected ALD, suspected significant fibrosis (F ≥ F2) was present in 12.3% (95% CI 4.74–28.34) and advanced fibrosis (F ≥ F3) was present in 6.31% (95% CI 0.69–39.55). Similarly, in subjects without excessive alcohol consumption, suspected NAFLD was present in 40.04% (36.64–43.54). In those with suspected NAFLD, suspected significant fibrosis (F ≥ F2) was present in 31.07% (27.25–35.16) and suspected advanced fibrosis (F ≥ F3) was present in 20.15% (16.05–24.99).

**Conclusion:**

A significant percentage of AYAs are at risk for ALD and NAFLD and a subset of these subjects is at risk for significant fibrosis. Efforts should focus on increasing awareness of the prevalence of ALD and NAFLD in this population and to mitigate modifiable risk factors.

**Supplementary Information:**

The online version contains supplementary material available at 10.1186/s12876-022-02430-7.

## Background

Non-alcoholic fatty liver disease (NAFLD) and alcoholic liver disease (ALD) are the two most common causes of chronic liver diseases and are characterized by the accumulation of excessive amounts of fat within the hepatocytes. NAFLD is currently considered as the most common cause of chronic liver disease worldwide and is the second leading cause of liver transplantation in the United States [1]. The prevalence of NAFLD is steadily and exponentially increasing with 25% of the adult population being affected in the year 2010 compared to only 15% in 2005. In a more recent cross-sectional study from 2011 to 2014, the overall prevalence of NAFLD among US adults was 21.9%, representing 51.6 million individuals affected, among which nearly 5 million (9.7%) had suspected advanced fibrosis [2].

Similarly, ALD counts for one of the very relevant, most prevalent and fastest growing liver diseases in the United States with an overall prevalence of 2%. Moreover, ALD is one of commonest causes of end-stage liver disease, with 50% of the overall mortality in cirrhosis patients being attributed to alcohol, and 50% of alcohol-related mortality being attributed to liver disease [3–5]. The increasing prevalence in NAFLD is mainly attributed to the large increase in the international prevalence of obesity and metabolic syndrome [6, 7], while the increasing prevalence of ALD is tightly linked to the worldwide increase in alcohol consumption as shown in the World Health Organization reports in 2016 [8].

Worrisomely, the demographic pattern of alcohol consumption has further shifted to the younger age groups resulting in a more substantial loss of productive life years and an increase in the alcohol-attributable mortality in the adolescents and young adults (AYAs), which is the age group most responsible for disability-adjusted life years (DALY) [8, 9].

The relationship between ALD and NAFLD is a two-sided and a rather complex one. For instance, numerous previous studies showed a possible protective effect of mild to moderate alcohol consumption on the development and progression of NAFLD [10–12], however; a more recent meta-analysis has shown the presence of possible confounders [13]. In fact, more recent studies have shown worse histological outcomes among NAFLD patients with moderate alcohol consumption [14, 15]. Similarly, a recent meta-analysis has suggested obesity as an independent risk factor for increased mortality among a specific ALD population [16].

Despite the significant prevalence and burden, the overall awareness of these two entities remains limited in the general population [17, 18]. Our aim is to assess the prevalence and burden of ALD and NAFLD in a representative cohort of AYAs in the United States by utilizing vibration-controlled transient elastography (VCTE) to directly measure hepatic steatosis and suspected fibrosis.

## Methods

### Database

The National Health and Nutrition Examination Survey (NHANES) database is a major project of the National Center for Health Statistics (NCHS) [19], which is affiliated with the Center for Disease Control and Prevention (CDC). The program is composed of subsets of studies that were designed to better understand the health and nutritional needs of children and adults in the United States and has been running for the last seven decades.

This survey-based program annually examines a nationally representative sample of ~ 5000 individuals located in counties across the United States. The NHANES interview includes demographic, socioeconomic, dietary, and health-related questions. The examination includes –but is not limited to- medical, physiological and laboratory measurements performed by highly trained medical personnel at a central laboratory, in addition to interview questionnaires and standardized physical examination.

Data from the survey is used in epidemiological and health-related studies and helps designing further health programs and services. The survey was approved by the Institutional Review Board at the Center for Disease Control and Prevention, and informed consent was obtained from all participants. Data from NHANES 2017–2018 is the most recent survey cycle that provided transient elastography information as determined by FibroScan^®^ and was utilized for this analysis.

### Definitions and inclusion criteria

**Fig. 1:**
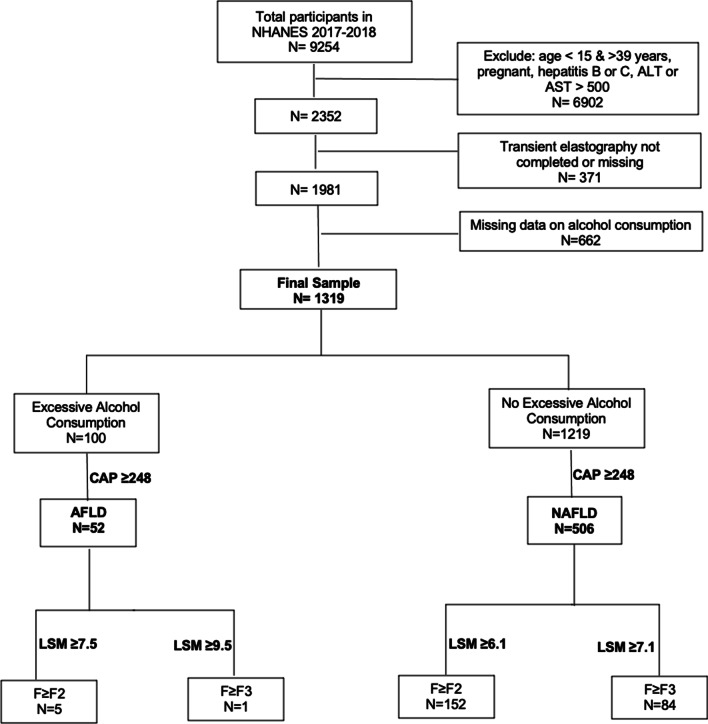
The inclusion criteria and participants stratification algorithm (also see Additional file 1)

Participants between 15 and 39 years with demographic (age, gender, ethnicity), alcohol intake data and a valid FibroScan^®^ were included in the analysis. Those with chronic viral hepatitis B or C, missing alcohol consumption data, pregnancy, Alanine Aminotransferase {ALT}, Aspartate Aminotransferase {AST} values > 500 U/L, or missing or incomplete elastography data were excluded. The inclusion criteria and participants stratification algorithm is shown in Fig. 1 (also see Additional file 1).

Daily alcohol consumption was calculated as: *Alcohol consumption* = *((average frequency of alcohol intake per year* × *average drinks per day))/(365 days)* using The Alcohol Use Questionnaire (ALQ) 130 {In the past 12 months, on those days that {you/SP} drank alcoholic beverages, on the average, how many drinks did {you/he/she} have?} and ALQ 121 {During the past 12 months, about how often did {you/SP} drink any type of alcoholic beverage? PROBE: How many days per week, per month, or per year did {you/SP} drink?} in the NHANES database. Excessive alcohol consumption was defined as male > 2 drinks/day and female > 1 drink/day, and the population was stratified into those with excessive alcohol consumption and those without. A “current smoker” was defined as any adult who has smoked 100 cigarettes in his/her lifetime and who currently smokes cigarettes. A “former smoker” was defined as any adult who has smoked at least 100 cigarettes in his/her lifetime but who had quit smoking at the time of interview. A “never smoker” was defined as any adult who has never smoked, or who has smoked less than 100 cigarettes in his/her lifetime. Controlled attenuation parameter (CAP) score ≥ 248 dB/m was used to define steatosis and to further stratify the population into suspected ALD and suspected NAFLD [20].

## Statistical analysis

### Characteristics of study participants

In addition to CAP score and liver stiffness measurement (LSM), the following socio-demographic information and laboratory data were collected for analysis: age, sex, race/ethnicity, alcohol intake status, smoking status (never smoked, former smoker, current smoker), Body Mass Index {BMI}, ALT, AST, total bilirubin, albumen, Mean Corpuscular Volume {MCV}, platelet count, and Haemoglobin A1C {HBA1C}.

### Outcome measures

In those with evidence of ALD, the cut-offs of LSM used for suspected fibrosis were F ≥ F2 at LSM ≥ 7.5 kPa and F ≥ F3 at ≥ 9.5 kPa, respectively. In those with suspected NAFLD, LSM cut-offs were F ≥ F2 at 6.1 and F ≥ F3 at ≥ 7.1, respectively. These cut-offs were chosen based on the published literature to maximize sensitivity [20–22]. We considered a 2-sided p value of < 0.05 as statistically significant. Chi-square test was used to compare categorical variables and t-test for continuous variables. Appropriate survey weights were applied for all analyses which were performed using Stata version 17 (StataCorp. 2021. *Stata Statistical Software: Release 17*. College Station, TX: StataCorp LLC.).

## Results

### Subject characteristics according to alcohol consumption

**Fig. 2:**
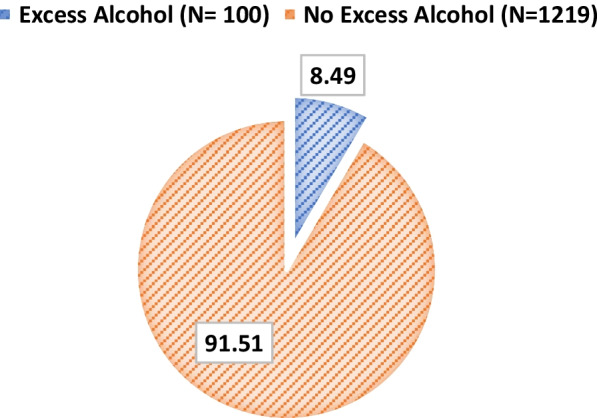
Percent prevalence excessive alcohol intake in the general population

**Fig. 3:**
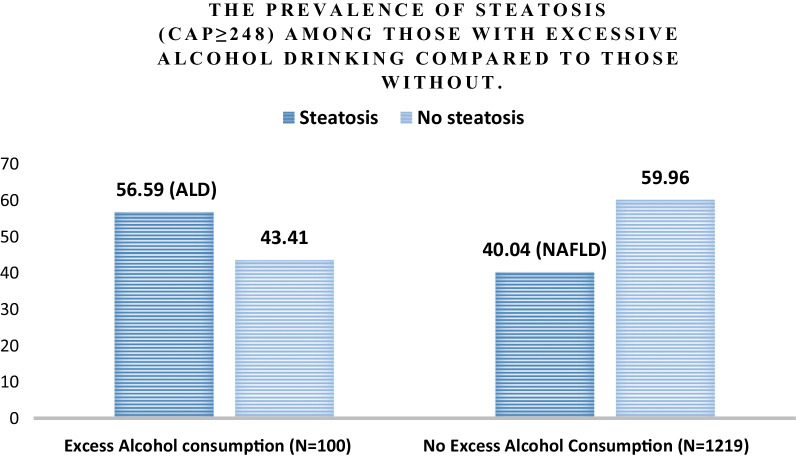
The Prevalence of steatosis (CAP ≥ 248) among those with excessive alcohol consumption compared to those without

Among the total number of noninstitutionalized civilian population screened in the database (N = 9254), our final sample consisted of 1319 AYAs after excluding those with viral hepatitis, pregnancy, ALT or AST > 500 U/L, missing data on alcohol consumption, and/or missing VCTE data. We reported the baseline characteristics of the study population (N = 1319), along with those with excessive alcohol consumption (N = 100) compared to those without (N = 1219) in Table 1. The prevalence of excessive alcohol consumption was estimated for the whole study population (Fig. 2). The prevalence of steatosis was then estimated for those with excessive alcohol consumption (i.e.: ALD prevalence) and compared to those without (i.e.: NAFLD prevalence) (Fig. 3). The characteristics both groups (ALD vs NAFLD) are shown in Table 2.

**Table 1:** Baseline characteristics of the study population

	Total	Excessive alcohol consumption	No excessive alcohol consumption	p value
	N = 1319	N = 100	N = 1219	
Age (years)—mean (95% CI)	28.63 (28.08–29.18)	29.82 (28.61–31.03)	28.52 (27.95–29.09)	0.049
Male—% (95% CI)	52.21 (47.94–56.46)	63.49 (53.12–72.74)	51.17 (46.3–56.02)	0.049
BMI (kg/m^2^)—mean (95% CI)	29.02 (28.01–30.02)	29.36 (27.89–30.83)	28.99 (27.96–30.02)	0.575
*Race/ethnicity*—*% (95% CI)*				
Non-Hispanic White	56.29 (49.71–62.66)	65.39 (55.99–73.72)	55.45 (48.53–62.16)	0.049
Non-Hispanic Black	12.19 (8.68–16.86)	9.19 (4.71–17.17)	12.47 (8.9–17.2)	
Hispanic	21.79 (16.35–28.42)	16.25 (9.47–26.45)	22.3 (16.72–29.09)	
Non-Hispanic Asian	4.77 (3.22–7.01)	1.31 (0.44–3.89)	5.09 (3.4–7.56)	
Non-Hispanic other	4.96 (3.68–6.66)	7.86 (3.88–15.29)	4.69 (3.31–6.62)	
*Smoking Status—% (95% CI)*				
Never	61.91 (55.82–67.65)	37.63 (22.42–55.76)	64.16 (57.64–70.2)	< 0.001
Former	17.3 (13.28–22.22)	11.33 (5.13–23.19)	17.85 (13.54–23.17)	
Current	20.79 (17.03–25.13)	51.03 (35.67–66.21)	17.99 (14.62–21.94)	
*Lab values*—*mean (95% CI)*				
Total bilirubin (mg/dL)	0.48 (0.44–0.52)	0.49 (0.39–0.6)	0.48 (0.44–0.52)	0.745
AST (IU/L)	22.47 (21.3–23.64)	28.19 (21.14–35.24)	21.94 (21.02–22.85)	0.064
ALT (IU/L)	24.72 (23–26.45)	32.27 (24.28–40.25)	24.03 (22.45–25.6)	0.038
Albumin (g/dL)	4.19 (4.14–4.25)	4.2 (4.13–4.26)	4.19 (4.13–4.25)	0.929
MCV (fL)	87.79 (87.14–88.43)	89.94 (88.47–91.41)	87.59 (86.94–88.24)	0.001
Platelet count (10^3^ cells/uL)	253.18 (246.33–260.02)	249.03 (234.65–263.42)	253.56 (246.49–260.63)	< 0.001
HbA1C (%)	5.3 (5.26–5.34)	5.22 (5.1–5.35)	5.31 (5.27–5.35)	0.002

**Table 2:** Characteristics of study participants with alcoholic liver disease in patients with excessive alcohol consumption compared to non-alcoholic fatty liver disease

	Alcoholic liver disease	Non-alcoholic fatty liver disease	p value
	N = 52	N = 506	
Age (years)—mean (95% CI)	30.02 (28.80–31.25)	30.00 (29.28–30.72)	0.968
Male—% (95% CI)	74.10 (56.42–86.34)	58.09 (50.92–64.92)	0.115
BMI (kg/m^2^)—mean (95% CI)	32.65 (30.98–34.31)	34.116 (32.80–35.43)	0.109
*Race/ethnicity*—*% (95% CI)*			
Non-Hispanic White	65.784 (51.21–77.89)	50.39 (41.47–59.28)	0.056
Non-Hispanic Black	5.368 (1.50–17.41)	9.56 (5.91–15.11)	
Hispanic	21.129 (12.96–32.52)	29.08 (21.19–38.49)	
Non-Hispanic Asian	0.554 (0.07–4.25)	6.14 (4.15–8.98)	
Non-Hispanic other	7.164 (2.29–20.29)	4.83 (3.23–7.16)	
*Smoking status—% (95% CI)*			
Never	26.66 (12.77–47.44)	59.88 (51.56–67.67)	< 0.001
Former	12.31 (4.83–27.98)	22.4 (16.56–29.56)	
Current	61.03 (45.18–74.85)	17.72 (12.97–23.74)	
*Lab values*—*mean (95% CI)*			
Total bilirubin (mg/dL)	0.45 (0.31–0.59)	0.46 (0.41–0.5)	0.905
AST (IU/L)	33.81 (21.67–45.95)	23.9 (22.4–25.4)	0.087
ALT (IU/L)	41.55 (26.48–56.62)	31.15 (27.29–35.01)	0.142
Albumin (g/dL)	4.2 (4.11–4.3)	4.14 (4.06–4.22)	0.379
MCV (fL)	89.8 (87.72–91.88)	86.34 (85.53–87.15)	0.002
Platelet count (10^3^ cells/uL)	260.16 (240.48–279.85)	265.58 (254.51–276.64)	0.591
HbA1C (%)	5.38 (5.13–5.63)	5.46 (5.37–5.55)	0.548

In the excessive alcohol consumption group, the characteristics of those with ALD were compared to those without in Table 3. Similarly, among the non-excessive alcohol consumption group, the characteristics of those with NAFLD were compared to those without liver involvement in Table 4.

**Table 3:** Characteristics of participants with alcoholic liver disease compared to those without in the excessive alcohol consumption group

	Alcoholic liver disease	No alcoholic liver disease	p value
	N = 52	N = 48	
Age (years)—mean (95% CI)	30.02 (28.80–31.25)	29.56 (26.53–32.58)	0.785
Male—% (95% CI)	74.10 (56.42–86.34)	49.65 (34.56–64.81)	0.044
BMI (kg/m^2^)—mean (95% CI)	32.65 (30.98–34.31)	25.16 (23.99–26.34)	< 0.001
*Race/ethnicity*—*% (95% CI)*			
Non-Hispanic White	65.784 (51.21–77.89)	64.87 (48.55–78.33)	0.256
Non-Hispanic Black	5.368 (1.50–17.41)	14.18 (5.91–30.3)	
Hispanic	21.129 (12.96–32.52)	9.88 (4.01–22.36)	
Non-Hispanic Asian	0.554 (0.07–4.25)	2.3 (0.58–8.71)	
Non-Hispanic other	7.164 (2.29–20.29)	8.77 (3.75–19.14)	
*Smoking status—% (95% CI)*			
Never	26.66 (12.77–47.44)	51.95 (29.78–73.37)	< 0.104
Former	12.31 (4.83–27.98)	10.05 (3.3–26.77)	
Current	61.03 (45.18–74.85)	38 (20.86–58.78)	
*Lab values*—*mean (95% CI)*			
Total bilirubin (mg/dL)	0.45 (0.31–0.59)	0.55 (0.42–0.68)	0.212
AST (IU/L)	33.81 (21.67–45.95)	20.91 (18.23–23.59)	0.062
ALT (IU/L)	41.55 (26.48–56.62)	20.24 (14.08–26.4)	0.042
Albumin (g/dL)	4.2 (4.11–4.3)	4.19 (4.09–4.28)	0.816
MCV (fL)	89.8 (87.72–91.88)	90.11 (88.17–92.06)	0.808
Platelet count (10^3^ cells/uL)	260.16 (240.48–279.85)	234.97 (218.29–251.64)	0.065
HbA1C (%)	5.38 (5.13–5.63)	5.02 (4.92–5.12)	0.015

**Table 4:** The characteristics of non-alcoholic fatty liver disease participants compared to those with no liver involvement in the non-excessive alcohol consumption group

	NAFLD	No NAFLD	p value
	N = 506	N = 713	
Age (years)—mean (95% CI)	30.00 (29.28–30.72)	27.53 (26.83–28.24)	< 0.001
Male—% (95% CI)	58.09 (50.92–64.92)	46.55 (40.47–52.74)	0.016
BMI (kg/m^2^)—mean (95% CI)	34.116 (32.80–35.43)	25.58 (24.85–26.3)	< 0.001
*Race/ethnicity*—*% (95% CI)*			
Non-Hispanic White	50.39 (41.47–59.28)	58.82 (51.21–66.04)	0.001
Non-Hispanic Black	9.56 (5.91–15.11)	14.41 (10.7–19.13)	
Hispanic	29.08 (21.19–38.49)	17.77 (13.15–23.57)	
Non-Hispanic Asian	6.14 (4.15–8.98)	4.39 (2.54–7.49)	
Non-Hispanic other	4.83 (3.23–7.16)	4.6 (2.8–7.48)	
*Smoking status—% (95% CI)*			
Never	59.88 (51.56–67.67)	67.02 (60.11–73.28)	0.054
Former	22.4 (16.56–29.56)	14.81 (10.25–20.93)	
Current	17.72 (12.97–23.74)	18.16 (14.29–22.8)	
*Lab values*—*mean (95% CI)*			
Total bilirubin (mg/dL)	0.46 (0.41–0.5)	0.49 (0.45–0.54)	0.172
AST (IU/L)	23.9 (22.4–25.4)	20.62 (19.02–22.23)	0.018
ALT (IU/L)	31.15 (27.29–35.01)	19.26 (17.78–20.73)	< 0.001
Albumin (g/dL)	4.14 (4.06–4.22)	4.23 (4.17–4.28)	0.020
MCV (fL)	86.34 (85.53–87.15)	88.43 (87.78–89.08)	< 0.001
Platelet count (10^3^ cells/uL)	265.58 (254.51–276.64)	245.45 (239.04–251.86)	< 0.001
HbA1C (%)	5.46 (5.37–5.55)	5.21 (5.18–5.24)	< 0.001

Finally, the prevalence and stage of suspected fibrosis for ALD and NAFLD were separately collected and compared in both groups.

The prevalence of excessive alcohol intake (N = 100) was 8.49% (95% CI 6.43–11.13) in the general population. In comparison to those without excessive alcohol consumption (N = 1219), subjects with excessive alcohol consumption (N = 100) were older (29.8 vs 28.5 years, p = 0.049), more likely to be male (63.4% vs 51.1%, p = 0.049), of Caucasians descendants (65.3% vs. 55.4%, p = 0.049), current smokers (51% vs. 17.9%, p < 0.001), had higher AST (28.2 vs. 21.9 U/L, p = 0.064), ALT (32.3 vs. 24 U/L, p = 0.038) and MCV (90 vs. 87.6 fL, p < 0.001), and had lower platelet counts (249 vs. 253.5 10^3^/uL p < 0.001) and HbA1C (5.22% vs. 5.31%, p = 0.002).

### The prevalence of ALD in AYAs

**Fig. 4:**
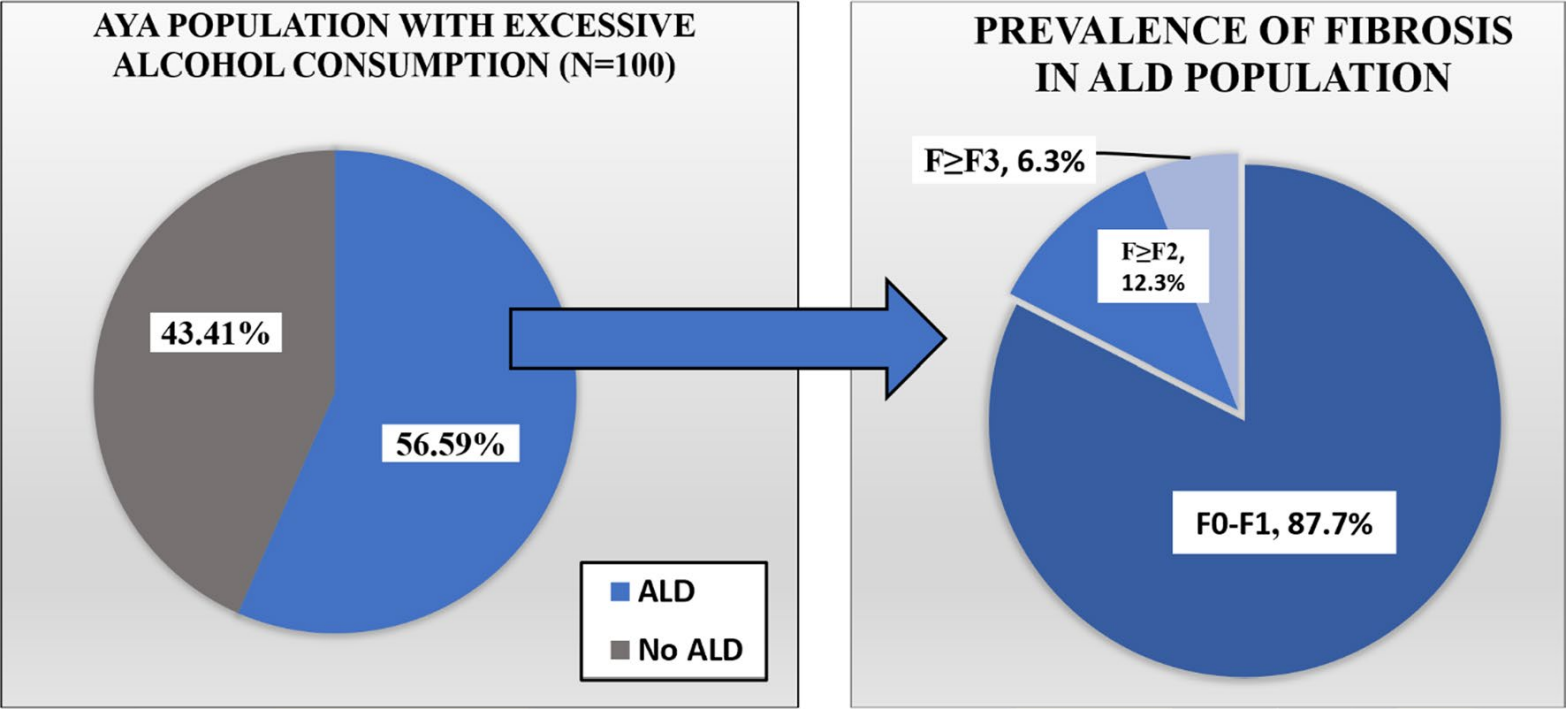
The prevalence and staging of alcoholic liver disease in adolescents and young adults with excessive alcohol consumption

Among those with excessive alcohol consumption, 56.59% (95% CI 41.57–70.49) had ALD compared to 43.41% (95% CI 29.51–58.43) who had no evidence of liver involvement based on CAP < 248 dB/m. The prevalence of suspected fibrosis in the ALD group was as follows (Fig. 4): F0–F1 87.7% (95% CI 71.66–95.26), F ≥ F2 12.3% (95% CI 4.74–28.34) and F ≥ F3 6.31% (95% CI 0.69–39.55).

Moreover, compared to those with no evidence of ALD based on low CAP score, subjects with ALD (N = 52) tended to be older (30.0 vs 26.5 years, p = 0.785), more males (74.1% vs 49.6%, p = 0.044), with higher BMI (32.6 vs 25.1 kg/m^2^, p ≤ 0.001), Caucasians (65.7 vs 64.8%, p = 0.256), current smokers (61.0% vs 38%, p ≤ 0.104), had higher AST (33.8 vs 20.9 IU/L, p = 0.062) and ALT (41.5 vs 20.2 IU/L, p = 0.042), comparable albumin (4.2 vs 4.19 g/dL, p = 0.816) and MCV (89.8 vs 90.1 fL, p = 0.808) levels, with higher platelet count (260.1 vs 234.9 10^3^ cells/uL, p = 0.065) and HbA1C (5.3% vs 5.0%, p = 0.015).

### The prevalence of NAFLD in AYAs

**Fig. 5:**
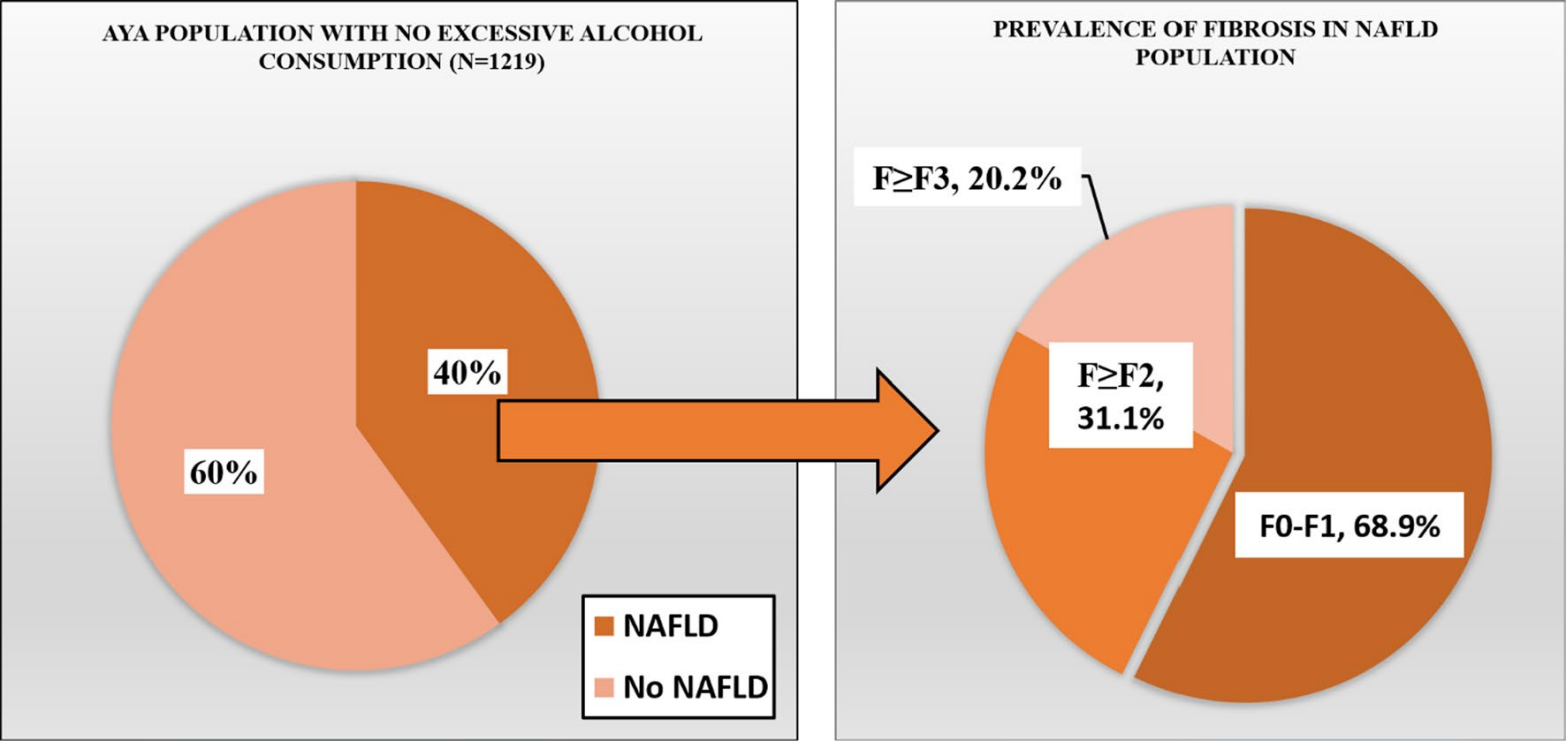
The prevalence and staging of non-alcoholic fatty liver disease in adolescents and young adults without excessive alcohol consumption

Among those with no excessive alcohol consumption (N = 1219), the prevalence of NAFLD was 40.04% (95% CI 36.64–43.54) compared to 59.96 (95% CI 56.46–63.36) who had no liver involvement. The prevalence of suspected fibrosis in the NAFLD group was as follows (Fig. 5): F0–F1 68.93% (95% CI 64.84–72.75), F ≥ F2 31.07 (95% CI 27.25–35.16) and F ≥ F3 20.15% (95% CI 16.05–24.99).

Also comparably, those with NAFLD (N = 506) tended to be older (30.0 vs 27.5 years, p < 0.001), more males (58.0% vs 46.5%, p = 0.016) than females, had significantly higher BMI (34.1 vs 25.5 kg/m^2^, p < 0.001), Hispanics (29.0% vs 17.7%, p = 0.001), former smokers (22.4 vs 14.8, p = 0.054), had higher AST (23.9 vs 20.6 IU/L, p = 0.018) and ALT (31.1 vs 19.2, p ≤ 0.001), lower albumin (4.1 vs 4.2 g/dL, p = 0.02), higher platelet counts (265.5 vs 245.4 10^3^ cells/uL, p < 0.001) and higher HbA1C (5.4% vs 5.2%, p < 0.001).

## Discussion

The main findings of this study are the following: (1) a significant percentage of AYAs consume excessive amounts of alcohol placing them at risk of ALD; (2) in those with excessive alcohol consumption, 57% have evidence of ALD based on CAP and 12% are at risk of having significant suspected fibrosis based on LSM; (3) in those without excessive alcohol consumption, 40% have evidence of NAFLD with 31% at risk of significant fibrosis; (4) male gender, obesity, smoking, and certain ethnicities were associated with higher burden of liver disease.

Unhealthy alcohol use and obesity are the two major drivers of the burden of fatty liver disease (FLD) across the globe [23, 24]. Alcohol is known to cause metabolic insult, but obesity-related liver diseases have grown from a scarcely known disease to a mounting epidemic over the last two decades. Evidently, in the twenty-first century, two things that have become more prevalent in AYA are unhealthy patterns of alcoholism and metabolic syndrome secondary to obesity. Several studies have stressed upon the entwining pathophysiology of NAFLD and ALD [25, 26]. We conducted a population-based cross-sectional retrospective study using a U.S representative sample from NHANES database 2017–2018 to screen AYAs using a valid FibroScan measurement. In our study, the prevalence of excessive alcohol intake was 8.49% (95% CI 6.43–11.13) in the general population. We demonstrated that a significant fraction of AYAs is at a higher risk for ALD and NAFLD (Figs. 4 and 5).

Compared to the most recently reported US prevalence of both diseases from the same database on 2008 cycle (age cut off: 18 or above), our results show a significantly higher prevalence for NAFLD (40.04% vs. 11.01%) and ALD (56% vs. 3.94%), confirming previously predicted exponential growth of the trends and burden of these two diseases [5]. The large difference between the two rates can be explained by the obesity and alcohol pandemics. However, the large difference seen in ALD rates can also be explained by the objectivity of our study in detecting the disease using a valid transient elastography results, and hence minimizing the underdiagnoses of this disease which has been historically underreported [4]. Furthermore, a subsection of AYA is at the risk of developing significant liver fibrosis. This study aimed to create awareness in AYA by assessing the prevalence and burden of ALD and NAFLD in a representative cohort of AYAs in the United States. In comparison to non-excessive alcohol consumption, AYAs with excessive alcohol consumption were found to be of Caucasians descendants (65.3% vs. 55.4%, p = 0.049), were current smokers (51% vs. 17.9%, p < 0.001), were older (29.8 vs 28.5 years, p = 0.049), and had higher BMI (29.2 vs. 27.6 kg/m^2^) with slight male predilection more (63.4% vs 51.1%, p = 0.049).

Ethnic factors play a role, as evidenced by epidemiological studies comparing different ethnic groups observing the rates of alcoholic cirrhosis. Ethnicity factors are postulated to play a role in liver metabolism via several genetic polymorphisms [27]. In our study prevalence of AYAs with excessive alcohol consumption was highest in the Caucasian race. This racial predilection can be postulated to population demographics and culture as explained in an epidemiological overview of the general population [28]. Another rationale for the increased risk of excessive alcohol intake in the Caucasian race could be linked to a low socioeconomic profile of various subgroups. A study by Willams and the group showed how low socioeconomic status is often related to disease burden and correlates to excessive alcohol consumption [29].

Although alcohol consumption is increasing in younger people more rapidly than ever, our study demonstrated that excessive alcohol intake is slightly higher in older individuals in the AYAs group. We believe that the addictive and cumulative nature of alcohol consumption is responsible for such findings.

Hart et al. did an exciting analysis showing how high BMI and alcohol consumption have a supra-additive interaction to cause liver disease [30]. Another study by Abeysekera demonstrated that raised BMI and adiposity were positively linked with increasing steatosis grade (p < 0·0001) [22].

Obesity is one of the most important modifiable risk factors which determines the risk of cirrhosis in heavy drinkers [30].

Current study also exhibits interesting comparative findings blood chemistry in AYAs. AYA with excessive alcohol consumption had higher AST (28.2 vs. 21.9 U/L, p = 0.064), ALT (32.3 vs. 24 U/L, p = 0.038) and MCV (90 vs. 87.6 fL, p < 0.001), and had lower platelet counts (249 vs. 253.5 10^3^/uL p < 0.001) and HbA1C (5.22% vs. 5.31%, p = 0.002). Hence, no unique pattern of liver enzymes or other markers has been seen.

Alcohol is the fourth leading preventable cause of death in the United States [31], and nearly 25% of the world’s population at present is thought to have been affected with NAFLD [32]. According to a metanalysis by Anderson et al., the pooled mean prevalence of NAFLD children from general population studies were 7.6% (95% CI 5.5–10.3%) and 34.2% (95% CI 27.8–41.2%) [33]. These numbers are still rising and can often lead to serious irreversible liver fibrosis and damage. There is a linear relationship between the liver injury rate and overall alcohol consumption [34, 35]. FLD comprises a broad spectrum of diseases ranging from asymptomatic or early disease to advanced pathologies. Thus, both alcohol and obesity, which are preventable risk factors for liver disease-associated mortality and morbidity, should be taken more seriously. Our aim through this paper was to provide a critical, comparative analysis of AYAs who are at risk of developing NAFLD and ALD.

An increase in inflammation (by alcohol or fatty acid oxidation) leads to an increase in stiffness, which causes irreversible scarring and fibrosis, thereby causing significant liver illnesses. Hepatic fibrosis has been established as a dynamic process with the potential for considerable resolution. Unlike an alcoholic liver disease, there is no unique elevation pattern of liver enzymes for diagnosing NAFLD.

We used transient electrography to quantify hepatic steatosis and suspected fibrosis. Due to considerable inter-observer variability, traditional USS imaging is not a very sensitive diagnostic tool to assess mild liver steatosis compared to liver biopsy [36]. The FDA has approved Vibration-Controlled Transient Elastography (FibroScan). This noninvasive diagnostic modality detects the degree of swelling, measures the degree of stiffness, and gives us a quantitative measure of liver steatosis [37, 38]. Fibroscan entails a CAP value that stands for controlled attenuation parameter, which provides a reasonable estimate of the degree of steatosis. The CAP score is a promising tool for the noninvasive detection of hepatic steatosis [39]. Another measurement in Fibroscan is called liver stiffness measurement (LSM), which is based on shear wave elastography. According to Siddiqui et al., a controlled attenuation parameter cutoff value of 270 dB/m detects any hepatic steatosis (95% CI 0.78–0.93) [40]. We used a cutoff of 248 dB/m to identify suspected ALD and suspected NAFLD in the two cohorts (with and without excessive alcohol consumption). Based on the CAP score after stratifying individuals into ALD and NAFLD, we used LSM to quantify suspected fibrosis. We used a LSM cutoff value > 7.5 kPa for F > F2 and 9.5 kPa for F > F3. Most of these cutoffs were chosen based on published literature to maximize the sensitivity [41].

Beyond these demographic results, our study extends the literature in a number of ways. First, we extricated the CAP score by Fibroscan and determined the suspected fibrosis occurrences in NAFLD and ALD individuals in our two cohorts of excessive alcohol and non-excessive alcohol consumption. While alcohol consumption is a well-thought-out cause of hepatic steatosis in the ALD process, NAFLD spans the spectrum of hepatic steatosis in the absence of alcohol consumption.

An epidemiological study by Abeysekera et al. had coherent conclusions to our findings. The participants with steatosis and alcohol use disorder were reported to have four times higher risk of developing fibrosis [22]. In the excessive alcohol consumption cohort, our results were consistent with the occurrence of 56.59% (95% CI 41.57–70.49) of individuals who were suspected of ALD (CAP score > 248 dB/m). These findings were consistent with another study by Niezen et al. [42]. However, in a recent study by Unalp-Arida A, which validated no association of heavy alcohol consumption development of steatosis [43].

In subjects without excessive alcohol consumption (N = 1219), suspected NAFLD was present in 40.04% (95% CI 36.64–43.54) of individuals.

In the fraction of individuals with suspected ALD, we found that for stage F0-F1 prevalence of suspected fibrosis was 87.7% (95% CI 71.66–95.26), for subsequent significant fibrosis stage (F ≥ F2) prevalence was 12.3% (95% CI 4.74–28.34), and advanced fibrosis stage F ≥ F3 6.31% (95% CI 0.69–39.55). For individuals with suspected NAFLD (N = 506), the prevalence of suspected fibrosis was stratified. For the stage F0-F1 prevalence was 68.93% (95% CI 64.84–72.75), for subsequent stage of suspected significant fibrosis F ≥ F2 prevalence was 31.07 (95% CI 27.25–35.16) and for suspected advanced fibrosis stage F ≥ F3 prevalence was 20.15% (95% CI 16.05–24.99).

These results were coherent with the findings observed in a community-based ABCD (Alimentazione, Benessere Cardiovascolare e Diabete) study of 890 individuals by Petta et al., which showed a stark high 60% prevalence of NAFLD in the general population and amongst NAFLD patients, there was a high prevalence of advanced fibrosis in specific variants (OR 3.06, 95% CI 1.08–8.65, P < 0.05) [44]. NAFLD was prevalent in 3·1% (28 of the 890 participants) and had evidence of advanced fibrosis (≥ F3).

### Limitations

Our study has several limitations. Firstly, despite using strict exclusion criteria, responses entail a degree of recall error considering the nature of the questionnaire for alcohol consumption history. This also leads to the generalizability of our findings to other patient populations requires clarification. For diagnosing NAFLD, we used the Fibroscan, which has limited sensitivity and validity to detect fibrosis equivalent to stage F2 or less [37, 45]. Since our data were collected from a national database, we also suspect the inter-system and interobserver variability for the measurement of CAP score and LSM is coherent with the findings of Ferraioli et al. [46]. We suspect that prevalence NAFLD could have been underreported in the AYAs group. Also, there is a significant overlap between NAFLD and ALD alcohol-related liver disease in many individuals in the AYAs subgroup. This may again make our cohort less generalizable to the standard population, especially in AYAs. This was also evident in a study by Long et al., where authors stressed the role of alcoholism in hepatic steatosis in patients with NAFLD [47]. Finally, in spite of the high certainty of representation of the general population in our initial cohort, how representative our sample was after applying the inclusion and exclusion criteria will remain unclear.

## Conclusion

Our study draws attention to the growing prevalence of FLD in AYA. Since there is limited evidence regarding understanding factors contributing to FLD in AYA, we need to focus more on mitigating these risk factors. Large randomized control trials are a curial necessity to have early and accurate detection of diffuse liver diseases in AYAs. Our study showed that a significant percentage of AYAs are at risk for ALD and NAFLD, and a fraction of these subjects are at risk of developing liver fibrosis. Efforts should focus on increasing awareness of the burden of ALD and NAFLD in this population and mitigate the modifiable risk factors. Educational programs, lifestyle interventions, and awareness are essential to acknowledge the prevalence of these two clinical entities, which are often intertwined in AYAs.

## Supplementary Information

**Additional file 1:** Numbers of excluded individuals among study participants with corresponding criteria.

## Data Availability

Datasets used in this analysis can be found online on NHANES database website (https://wwwn.cdc.gov/nchs/nhanes/) for free public access.

## Abbreviations

NAFLD: Non-alcoholic liver disease
ALD: Alcoholic liver disease
AYAs: Adolescents and young adults
DALY: Disability-adjusted life years
VCTE: Vibration-controlled transient elastography
NHANES: The National Health and Nutrition Examination Survey
NCHS: National Center for Health Statistics
CDC: Center for Disease Control and Prevention
ALQ: The Alcohol Use Questionnaire
LSM: Liver stiffness measurement
CAP: Controlled attenuation parameter
ALT: Alanine Aminotransferase
AST: Aspartate Aminotransferase
BMI: Body Mass Index
MCV: Mean corpuscular volume
HBA1C: Haemoglobin A1C
FLD: Fatty liver disease

## References

[1] Younossi Z, Anstee QM, Marietti M, Hardy T, Henry L, Eslam M, George J, Bugianesi E (2018). Global burden of NAFLD and NASH: trends, predictions, risk factors and prevention. Nat Rev Gastroenterol Hepatol.

[2] Wong RJ, Liu B, Bhuket T (2017). Significant burden of nonalcoholic fatty liver disease with advanced fibrosis in the US: a crosssectional analysis of 2011–2014 National Health and Nutrition Examination Survey. Aliment Pharmacol Ther.

[3] Asrani SK, Devarbhavi H, Eaton J, Kamath PS (2019). Burden of liver diseases in the world. J Hepatol.

[4] Rehm J, Samokhvalov AV, Shield KD (2013). Global burden of alcoholic liver diseases. J Hepatol.

[5] Younossi ZM, Stepanova M, Afendy M, Fang Y, Younossi Y, Mir H, Srishord M (2011). Changes in the prevalence of the most common causes of chronic liver diseases in the United States from 1988 to 2008. Clin Gastroenterol Hepatol.

[6] Wieland AC, Mettler P, McDermott MT, Crane LA, Cicutto LC, Bambha KM (2015). Low awareness of nonalcoholic fatty liver disease among patients at high metabolic risk. J Clin Gastroenterol.

[7] Mozumdar A, Liguori G (2011). Persistent increase of prevalence of metabolic syndrome among U.S. adults: NHANES III to NHANES 1999–2006. Diabetes Care.

[8] WHO (2018). Global status report on alcohol and health 2018.

[9] Review for "Global burden and attributable risk factors of acute lymphoblastic leukemia in 204 countries and territories in 1990–2019: estimation based on global burden of disease study 2019". 2021. 10.1002/hon.2936/v2/review1 10.1002/hon.2936 34664286

[10] Ajmera VH, Terrault NA, Harrison SA (2017). Is moderate alcohol use in nonalcoholic fatty liver disease good or bad? A critical review. Hepatology.

[11] Moriya A, Iwasaki Y, Ohguchi S, Kayashima E, Mitsumune T, Taniguchi H, Ikeda F, Shiratori Y, Yamamoto K (2010). Alcohol consumption appears to protect against non-alcoholic fatty liver disease. Aliment Pharmacol Therap.

[12] Dunn W, Sanyal AJ, Brunt EM, Unalp-Arida A, Donohue M, McCullough AJ, Schwimmer JB (2012). Modest alcohol consumption is associated with decreased prevalence of steatohepatitis in patients with non-alcoholic fatty liver disease (NAFLD). J Hepatol.

[13] Ajmera V, Belt P, Wilson LA, Gill RM, Loomba R, Kleiner DE, Neuschwander-Tetri BA, Terrault N (2018). Among patients with nonalcoholic fatty liver disease, modest alcohol use is associated with less improvement in histologic steatosis and steatohepatitis. Clin Gastroenterol Hepatol.

[14] Mehta M, Satsangi S, Duseja A, Taneja S, Dhiman RK, Chawla YK (2016). Can alcoholic liver disease and nonalcoholic fatty liver disease Co-exist?. J Clin Exp Hepatol.

[15] Mehta M, Satsangi S, Duseja A, Taneja S, Dhiman RK, Chawla Y (2017). Can alcoholic liver disease and nonalcoholic fatty liver disease co-exist?. J Clin Exp Hepatol.

[16] Chamorro A, Torres J, Mirón-Canelo J, González-Sarmiento R, Laso F, Marcos M (2014). Systematic review with meta-analysis: the I148M variant of patatin-like phospholipase domain-containing 3 gene (PNPLA3) is significantly associated with alcoholic liver cirrhosis. Aliment Pharmacol Therap.

[17] Singh A, Dhaliwal AS, Singh S, Kumar A, Lopez R, Gupta M, Noureddin M, Carey W, McCullough A, Alkhouri N (2019). Awareness of nonalcoholic fatty liver disease is increasing but remains very low in a representative US cohort. Dig Dis Sci.

[18] Jun DW, Cho YK, Sohn JH, Lee CH, Kim SH, Eun JR (2011). A study of the awareness of chronic liver diseases among Korean adults. Korean J Hepatol.

[19] National Center for Health Statistics (US) (2019). Health, United States, 2018.

[20] Karlas T, Petroff D, Sasso M, Fan J, Mi Y, De Lédinghen V, Kumar M, Lupsor-Platon M, Han K, Cardoso AC, Ferraioli G, Chan W, Wong VW, Myers RP, Chayama K, Friedrich-Rust M, Beaugrand M, Shen F, Hiriart J, Sarin SK, Badea R, Jung KS, Marcellin P, Filice C, Mahadeva S, Wong GL-H, Crotty P, Masaki K, Bojunga J, Bedossa P, Keim V, Wiegand J (2017). Individual patient data meta-analysis of controlled attenuation parameter (CAP) technology for assessing steatosis. J Hepatol.

[21] Fourth national report on human exposure to environmental chemicals. Updated tables, March 2021: volume two: NHANES 2011–2016; 2021. 10.15620/105345

[22] Abeysekera KW, Fernandes GS, Hammerton G, Portal AJ, Gordon FH, Heron J, Hickman M (2020). Prevalence of steatosis and fibrosis in young adults in the UK: a population-based study. Lancet Gastroenterol Hepatol.

[23] Chalasani N, Younossi Z, Lavine JE, Diehl AM, Brunt EM, Cusi K, Charlton M, Sanyal AJ (2012). The diagnosis and management of non-alcoholic fatty liver disease: Practice guideline by the American Association for the Study of Liver Diseases, American College of Gastroenterology, and the American Gastroenterological Association. Hepatology.

[24] Younossi Z, Henry L (2016). Contribution of alcoholic and nonalcoholic fatty liver disease to the burden of liver-related morbidity and mortality. Gastroenterology.

[25] Idalsoaga F, Kulkarni AV, Mousa OY, Arrese M, Arab JP (2020). Non-alcoholic fatty liver disease and alcohol-related liver disease: two intertwined entities. Front Med.

[26] Ntandja Wandji LC, Gnemmi V, Mathurin P, Louvet A (2020). Combined alcoholic and non-alcoholic steatohepatitis. JHEP Rep.

[27] Agarwal D (2001). Genetic polymorphisms of alcohol metabolizing enzymes. Pathol Biol (Paris).

[28] Hasin D, Delker E (2015). The National Epidemiologic Survey on Alcohol and Related Conditions (NESARC)—a huge resource for data and research findings. Addiction.

[29] Williams R, Alexander G, Armstrong I, Baker A, Bhala N, Camps-Walsh G, Cramp ME, Lusignan S, Day N, Dhawan A, Dillon J, Drummond C, Dyson J, Foster G, Gilmore I, Hudson M, Kelly D, Langford A, McDougall N, Meier P, Moriarty K, Newsome P, O'Grady J, Pryke R, Rolfe L, Rice P, Rutter H, Sheron N, Taylor A, Thompson J, Thorburn D, Verne J, Wass J, Yeoman A (2018). Disease burden and costs from excess alcohol consumption, obesity, and viral hepatitis: Fourth report of the Lancet standing Commission on liver disease in the UK. Lancet.

[30] Hart CL, Morrison DS, Batty GD, Mitchell RJ, Davey Smith G (2010). Effect of body mass index and alcohol consumption on liver disease: analysis of data from two prospective cohort studies. BMJ.

[31] Ye Y, Kerr WC (2010). Alcohol and liver cirrhosis mortality in the United States: comparison of methods for the analyses of time-series panel data models. Alcohol Clin Exp Res.

[32] Mrad RA, Merjaneh N, Mubarak G, Lopez R, Zein NN, Alkhouri N (2016). The increasing burden of nonalcoholic fatty liver disease among young adults in the United States: a growing epidemic. Hepatology.

[33] Anderson EL, Howe LD, Jones HE, Higgins JP, Lawlor DA, Fraser A (2015). The prevalence of non-alcoholic fatty liver disease in children and adolescents: a systematic review and meta-analysis. PLoS ONE.

[34] Stein E, Cruz-Lemini M, Altamirano J, Ndugga N, Couper D, Abraldes JG, Bataller R (2016). Heavy daily alcohol intake at the population level predicts the weight of alcohol in cirrhosis burden worldwide. J Hepatol.

[35] Sheron N (2016). Alcohol and liver disease in Europe—simple measures have the potential to prevent tens of thousands of premature deaths. J Hepatol.

[36] Hernaez R, Lazo M, Bonekamp S, Kamel I, Brancati FL, Guallar E, Clark JM (2011). Diagnostic accuracy and reliability of ultrasonography for the detection of fatty liver: a meta-analysis. Hepatology.

[37] Wong VW, Vergniol J, Wong GL, Foucher J, Chan HL, Le Bail B, Choi PC, Kowo M, Chan AW, Merrouche W, Sung JJ, De Lédinghen V (2009). Diagnosis of fibrosis and cirrhosis using liver stiffness measurement in nonalcoholic fatty liver disease. Hepatology.

[38] Foucher J (2006). Diagnosis of cirrhosis by transient elastography (FibroScan): A prospective study. Gut.

[39] Myers RP, Pollett A, Kirsch R, Pomier-Layrargues G, Beaton M, Levstik M, Duarte-Rojo A, Wong D, Crotty P, Elkashab M (2012). Controlled attenuation parameter (CAP): a noninvasive method for the detection of hepatic steatosis based on transient elastography. Liver Int.

[40] Siddiqui MS, Idowu MO, Stromberg K, Sima A, Lee E, Patel S, Ghaus S, Driscoll C, Sterling RK, John B, Bhati CS (2021). Diagnostic performance of vibration-controlled transient elastography in liver transplant recipients. Clin Gastroenterol Hepatol.

[41] Newsome PN, Sasso M, Deeks JJ, Paredes A, Boursier J, Chan W, Yilmaz Y, Czernichow S, Zheng M, Wong VW, Allison M, Tsochatzis E, Anstee QM, Sheridan DA, Eddowes PJ, Guha IN, Cobbold JF, Paradis V, Bedossa P, Miette V, Fournier-Poizat C, Sandrin L, Harrison SA, Harrison SA (2020). FibroScan-AST (FAST) score for the non-invasive identification of patients with non-alcoholic steatohepatitis with significant activity and fibrosis: A prospective derivation and global validation study. Lancet Gastroenterol Hepatol.

[42] Niezen S, Trivedi HD, Mukamal KJ, Jiang ZG (2021). Associations between alcohol consumption and hepatic steatosis in the USA. Liver Int.

[43] Unalp-Arida A, Ruhl CE (2020). Transient elastography assessed hepatic steatosis and fibrosis are associated with body composition in the United States. Clin Gastroenterol Hepatol.

[44] Petta S, Di Marco V, Pipitone RM, Grimaudo S, Buscemi C, Craxì A, Buscemi S (2018). Prevalence and severity of nonalcoholic fatty liver disease by transient elastography: genetic and metabolic risk factors in a general population. Liver Int.

[45] Eddowes PJ, Sasso M, Allison M, Tsochatzis E, Anstee QM, Sheridan D, Guha IN, Cobbold JF, Deeks JJ, Paradis V, Bedossa P, Newsome PN (2019). Accuracy of FibroScan controlled attenuation parameter and liver stiffness measurement in assessing steatosis and fibrosis in patients with nonalcoholic fatty liver disease. Gastroenterology.

[46] Ferraioli G, De Silvestri A, Lissandrin R, Maiocchi L, Tinelli C, Filice C, Barr R (2018). Evaluation of inter-system variability in liver stiffness measurements. Ultraschall Medizin Eur J Ultrasound.

[47] Long MT, Massaro JM, Hoffmann U, Benjamin EJ, Naimi TS (2020). Alcohol use is associated with hepatic steatosis among persons with presumed nonalcoholic fatty liver disease. Clin Gastroenterol Hepatol.

[48] Alkhouri N, Le P, Yang J, Sakkal C, Polanco P, Vos M, Noureddin M (2021). The prevalence of alcoholic and nonalcoholic fatty liver disease in adolescents and young adults in the United States: analysis of the NHANES database. Oral Abstr Hepatol.

