# Supplementary material for: The prevalence of alcoholic and nonalcoholic fatty liver disease in adolescents and young adults in the United States: analysis of the NHANES database
Source: BMC Gastroenterol. 2022 Jul 30;22:366. doi: 10.1186/s12876-022-02430-7 (PMC9338651; doi:10.1186/s12876-022-02430-7)
Supplement: Supplementary file 1 — Additional file 1: Numbers of excluded individuals among study participants with corresponding criteria. [file 12876_2022_2430_MOESM1_ESM.docx]

**Supplementary material**

| **NHANES 2017-2018 (N=9254)** | 9254 |  |  |  |  |
| --- | --- | --- | --- | --- | --- |
|  |  | Not Aged 15-39 | 6840 | Complete FibroScan | 1981 |
|  |  | Pregnant | 51 | Partial complete | 153 |
|  |  | Hep B or C | 11 | Ineligible | 56 |
|  |  | AST or ALT >500 | 0 | Not done | 27 |
|  |  |  |  | Missing | 135 |
| **Exclude Total** |  |  | 6902 |  | 371 |
| **Missing alcohol data** | 662 |  |  |  |  |
| **Final sample** | **1319** |  |  |  |  |
